# Supplementary material for: Characterization of the Relationship between the Chaperone and Lipid-Binding Functions of the 70-kDa Heat-Shock Protein, HspA1A
Source: Int J Mol Sci. 2020 Aug 20;21(17):5995. doi: 10.3390/ijms21175995 (PMC7503672; doi:10.3390/ijms21175995)
Supplement: Supplementary file 1 [file ijms-21-05995-s001.pdf]

Characterization of the relationship between the chaperone and lipid-binding functions of the 70-kDa heat-shock protein, HspA1A

Larissa Smulders[1], Amanda J Daniels[1], Caroline B Plescia[2], Devon Berger[1], Robert V Stahelin[2], Nikolas Nikolaidis[1]*

[1] Department of Biological Science, Center for Applied Biotechnology Studies, and Center for Computational and Applied Mathematics, College of Natural Sciences and Mathematics, California State University Fullerton, Fullerton, CA 92834-6850, USA

[2] Department of Medicinal Chemistry and Molecular Pharmacology and the Purdue University Center for Cancer Research, Purdue University, West Lafayette, IN 47907, USA

*Corresponding author: Nikolas Nikolaidis; Department of Biological Science, Center for Applied Biotechnology Studies, and Center for Computational and Applied Mathematics, College of Natural Sciences and Mathematics, California State University Fullerton, Fullerton, CA 92834-6850; Tel: 657-278-4526; e-mail: nnikolaidis@fullerton.edu)

Supplementary Information

Supplementary Table 1

Supplementary Fig. 1

Supplementary Table 1 Statistical analyses of the experiments shown in Figures 3 and 4.

**ATPase Rate (Fig. 3)**			
	**p-values**		
**Pairs**	**0-30**	**0-60**	**0-90**
HspA1A-HspA1A+POPC	0.005857	0.008295	0.001005
HspA1A-HspA1A+POPS	0.013538	0.001005	0.001005
HspA1A+POPC-HspA1A+POPS	0.702006	0.107585	0.521428
HspA1A-HspA1A+DPPC	0.001005	0.001005	0.001005
HspA1A-HspA1A+DPPS	0.001005	0.001005	0.001005
HspA1A+DPPC-HspA1A+DPPS	0.36817	0.282109	0.899995
**Refolding Rate (Fig. 4)**			
**Pairs**	**p-value**		
HspA1A-HspA1A+POPC	0.69703		
HspA1A-HspA1A+POPS	0.00548		
HspA1A-HspA1A+DPPC	0.24448		
HspA1A-HspA1A+DPPS	0.00101		
HspA1A+POPC-HspA1A+POPS	0.00101		
HspA1A+POPS-HspA1A+DPPS	0.67417		
HspA1A+DPPC-HspA1A+DPPS	0.02169		

Supplementary Fig. 1 HspA1A binds to liposomes containing phosphatidylserine. A single concentration of liposomes (1 mM total lipid) composed of different lipid mixtures in specified molar ratio (see figure) was incubated with 1 μM of protein. After centrifugation, the extent of lipid-protein binding was determined by comparing the amount of protein that remained in the supernatant to the amount of protein that was pelleted. Representative SDS-PAGE gel electrophoresis of the supernatant (S) and pellet (P) fractions of HspA1A (two separate batches) with liposomes composed of different PC and PS lipid species.

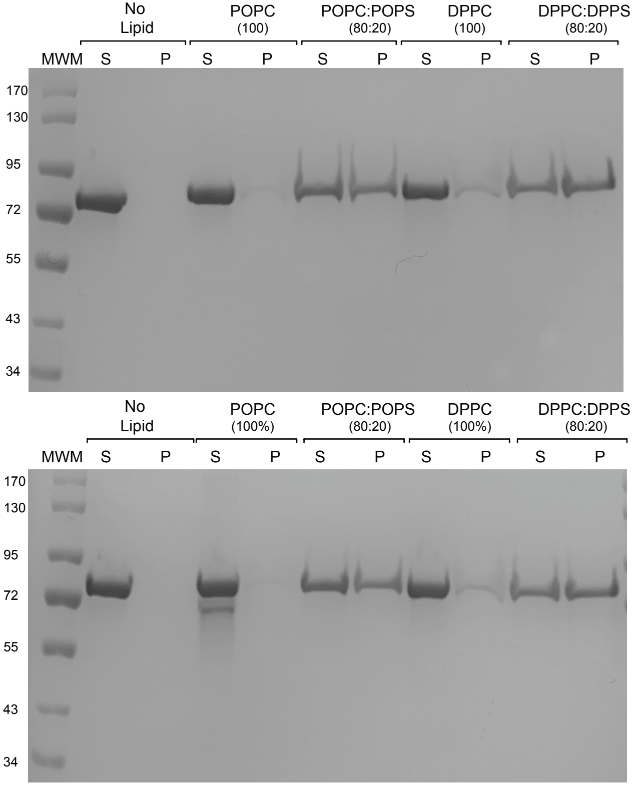

No Lipid
POPC (100)
POPC:POPS (80:20)
DPPC (100)
DPPC:DPPS (80:20)
MWM S P S P S P S P S P
170
130
95
72
55
43
34
No Lipid
POPC (100%)
POPC:POPS (80:20)
DPPC (100%)
DPPC:DPPS (80:20)
MWM S P S P S P S P S P
170
130
95
72
55
43
34
